# Early Tendon Morphology as a Biomarker of Long-term Patient Outcomes After Surgical Repair of Achilles Tendon Rupture: A Prospective Cohort Study

**DOI:** 10.1177/23259671231205326

**Published:** 2023-11-06

**Authors:** Annukka Saarensilta, Susanna Aufwerber, Karin Grävare Silbernagel, Paul Ackermann

**Affiliations:** † Department of Molecular Medicine and Surgery, Karolinska Institutet, Stockholm, Sweden; ‡ Women’s Health and Allied Health Professionals Theme, Medical Unit Occupational Therapy and Physiotherapy, Karolinska University Hospital, Stockholm, Sweden; § Department of Physical Therapy, University of Delaware, Newark, Delaware, USA; ‖ Department of Trauma, Acute Surgery and Orthopaedics, Karolinska University Hospital, Stockholm, Sweden; Investigation performed at Karolinska Institutet, Karolinska University Hospital, Stockholm, Sweden

**Keywords:** Achilles tendon rupture, tendon elongation, cross-sectional area, biomarker, patient outcome

## Abstract

**Background:**

Patient outcome after acute Achilles tendon rupture (ATR) varies and is difficult to predict. Whether early variations in healing, visualized with ultrasonography, can predict long-term patient outcome is unclear.

**Purpose:**

To (1) examine the associations of Achilles tendon cross-sectional area (CSA) and elongation (TE) during healing of ATR repair with patient outcomes at 12 months postoperatively and (2) investigate the predictive or diagnostic capacity of the morphological biomarkers.

**Study Design:**

Cohort study; Level of evidence, 2.

**Methods:**

This study was based on previously collected data from 86 patients who underwent acute standardized ATR repair between 2013 and 2018 and who were included in a prior randomized trial investigating early functional mobilization (EFM). In the EFM group, loading was allowed immediately after surgery, while in the comparison group, loading was allowed first at 2 weeks postoperatively. Achilles tendon CSA and length were measured with ultrasound at 6 weeks, 6 months, and 12 months postoperatively. CSA ratio and absolute difference in the length of the healthy and injured tendons were calculated. Patient-reported outcome was registered with the validated Achilles tendon Total Rupture Score and functional outcome with the heel-rise endurance test at 12 months postoperatively. The limb symmetry index (LSI) was calculated for maximum heel-rise height (HRH_max_) and total concentric work. Multiple linear regression adjusted for age was used, and the area under the receiver operating characteristic curve (AUC) was calculated to evaluate predictive capacity.

**Results:**

A larger CSA ratio at 6 weeks was associated with higher LSI HRH_max_ at 12 months (*R*^2^, 0.35; *P* < .001) and exhibited good predictive capacity (AUC, 0.82). More TE at 12 months was associated with lower LSI total concentric work at 12 months (*R*^2^, 0.21; *P* = .001) and exhibited acceptable predictive capacity (AUC, 0.71).

**Conclusion:**

Greater Achilles tendon CSA seen on ultrasound 6 weeks after surgical repair had good clinical prediction for long-term functional outcome. TE at 12 months was predictive of inferior functional outcome.

**Registration:**

NCT02318472 (ClinicalTrials.gov identifier).

Long-term functional limitations are common after acute Achilles tendon rupture (ATR), and prediction of patient outcome is a major challenge.^10,19,20^ Significant hereditary and acquired individual variations in healing response are expected to influence tendon morphology (ie, form and size) during tissue repair.^1,30^ Different methods have been applied to assess Achilles tendon dimensions,^1,6,11-13,16,17,21,23^ but ultrasound (US) has the best clinical applicability and is considered reliable.^26^ However, only a few studies^24,30,31^ have directly investigated early US measurements of Achilles tendon morphology as a predictive biomarker of long-term patient outcomes of ATR, which could be utilized in clinical practice and in research when developing healing optimizing treatments.

The early proliferative healing phase post-ATR repair provides a callus to stabilize the ruptured area.^29^ The size of the callus can be assessed by the tendon cross-sectional area (CSA) using US.^1,11,30,31^ A remodeling phase starts a couple of months after injury and lasts for more than a year, while the size of the callus successively decreases as the mechanical properties increase.^1,11,29^ It is unclear if tendon CSA during early healing is a good predictor of long-term patient outcome.

Tendon length (TL) is, in addition to the size of the callus, affected by the ATR healing process.^1,5^ Tendon elongation (TE) starts during early healing followed by a later modest shortening^1,5,12^ and may contribute to long-term impairments in tendon biomechanics.^5^ A systematic review published in 2020 showed heterogeneous methods in measuring TE and concluded insufficient evidence regarding TE as a biomarker of clinical patient outcomes.^5^ In a more recent study, TE >3 cm at 6 months was associated with inferior functional outcome at 12 months.^31^ Thus, whether TE can predict long-term outcome is not yet clearly established.

The primary aim of this study was to assess associations between US measurements of Achilles tendon morphology at 6 weeks, 6 months, and 12 months with patient outcomes at 12 months after surgery. The secondary aim was to investigate the predictive or diagnostic capacity of the morphological biomarkers. It was hypothesized that morphological biomarkers would serve as reliable, noninvasive ways to predict long-term patient outcomes.

## Methods

### Study Population

This was a prospective cohort study based on a randomized controlled trial (RCT) comparing early functional mobilization (EFM) with traditional plaster cast followed by orthosis after surgical repair of ATR.^2^ Patients were recruited from 3 hospitals (Karolinska University Hospital, Danderyd Hospital, and Södersjukhuset) in Stockholm between 2013 and 2018, with all evaluations performed at Karolinska University Hospital. The study protocol received institutional review board approval, and all patients provided written informed consent. The RCT was registered at ClinicalTrials.gov.^2^

Inclusion criteria applied in the RCT were 18 to 75 years of age, an acute unilateral ATR, and surgery performed within 1 week. For the purposes of this analysis, a further inclusion criterion—participation in tendon morphology measurements during ≥1 time point—was applied. Exclusion criteria were current anticoagulation therapy, heart failure with pitting edema, known kidney failure thromboembolic event during the previous 3 months, thrombophlebitis, known malignancy, pregnancy, hemophilia, other surgery during the previous month, inability to follow instructions, and planned follow-up at another hospital.^2^

**Figure 1.**
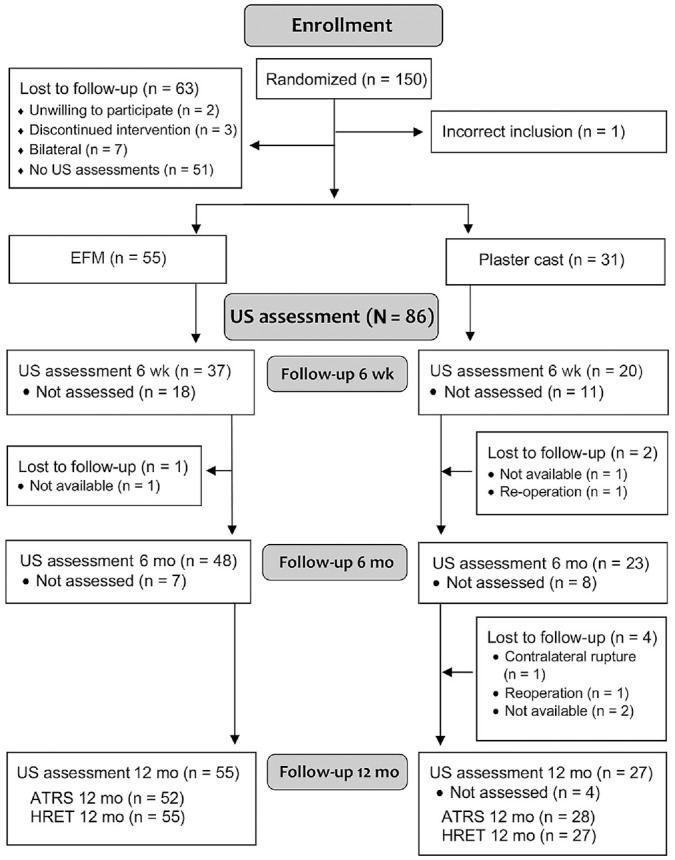
CONSORT (Consolidated Standards of Reporting Trials) flowchart of patient selection. ATRS, Achilles tendon Total Rupture Score; EFM, early functional mobilization; HRET, heel-rise endurance test; US, ultrasound.

US measurements of tendon morphology were performed for a total of 86 patients by a single experienced physiotherapist (S.A.). Not all patients participated in US measurements at all time points (Figure 1). The characteristics of the 86 patients who participated in US evaluation at any time point are presented in Table 1.

**Table 1:** Characteristics of Patients Included in the Study (N = 86)

Characteristic	Value
Age, y	39 ± 8.2
Sex, male	66 (77)
BMI, kg/m^2^	25 ± 2.7
Snuff use	19 (22)
Time from injury to surgery, h	99 ± 42
Surgery duration, h (n = 82)	0:48 ± 0:17
Postoperative treatment	
Plaster cast	31 (36)
EFM	55 (64)
SSI (n = 81)	2 (2.3)
DVT	29 (34)
Rerupture	0 (0)

Data are presented as mean ± SD or n (%). BMI, body mass index; DVT, deep venous thrombosis; EFM, early functional mobilization; SSI, surgical-site infection.

### Surgical Procedure

All patients underwent surgery using a predefined standardized surgical technique including a modified Kessler suture with two 1-0 polydioxanone sutures (Ethicon) as described previously.^2^ Paratenon and fascial cruris were sutured separately with 3-0 vicryl sutures (Ethicon).^2^

### Postoperative Treatment

In the RCT, patients were randomly allocated for the first 6 postoperative weeks to standard care in a plaster cast for 2 weeks followed by orthosis (Aircast AirSelect Elite; DJO) or to EFM in ankle-mobile orthosis (VACOped; OPED).^2^ Randomization was performed in a 2:1 ratio so that more patients were allocated to the intervention group (ie, EFM).^2^

In the intervention group, VACOped allowed initially a range of motion of 15° to 30° of plantarflexion and was increased to 5° to 30° after 2 weeks.^2^ Full weightbearing was allowed immediately. In the control group, a plaster cast in 30° equinus position was applied, and no weightbearing was allowed during the first 2 weeks.^2^ After 2 weeks, an ankle stabile orthosis (Aircast AirSelect Elite), and three 11-mm heel wedges were applied, providing 22° equinus; 1 wedge per week was gradually removed.^2^

At 2 weeks postoperatively, both groups were advised to perform unloaded plantarflexion exercises from a neutral position with a free degree of plantarflexion as well as ankle circles in plantarflexion. Stationary bicycle workouts were also allowed with orthosis. The exercises were performed at home, and no individual physical therapy was applied during the first 6 weeks.^3^ Immobilization concluded in both groups at 6 weeks postoperatively, whereafter a 1-cm heel wedge was worn in normal shoes for another 4 weeks. All study participants received a suggested rehabilitation program and were encouraged to contact a physical therapist in primary care for further supervised rehabilitation.^2^

### US Assessment

**Figure 2.**
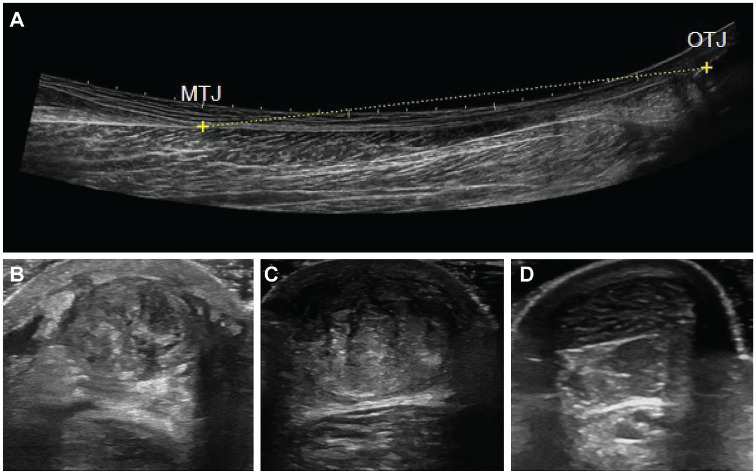
Ultrasound images of Achilles tendon length and cross-sectional area. (A) Length of a healthy Achilles tendon. (B) Cross-sectional area of an injured Achilles tendon at 6 weeks. (C) Cross-sectional area of an injured Achilles tendon at 6 months. (D) Cross-sectional area of a healthy Achilles tendon. MTJ, musculotendinous junction; OTJ, osteotendinous junction.

US measurements were performed at 6 weeks, 6 months, and 12 months by 1 experienced physical therapist (S.A.) as described previously.^1^ A wide-band linear array probe (5.0-12.0 MHz; GE Logiq e; GE Healthcare) in B mode at 10 MHz was used.^1^ Patients were asked to lie on an examination table in a prone position, with their feet hanging over the edge. Achilles TL was measured from the gastrocnemius myotendinous junction (MTJ) to the proximal insertion on the calcaneal bone, i.e. the osteotendinous junction (OTJ) using extended field-of-view (EFOV) settings (GE Logiq e; GE Healthcare) with the straight-line measurement tool (Figure 2).^1^ EFOV US has been reported to be a valid method for Achilles TL evaluation, and the test-retest reliability and interrater reliability have been reported to be excellent.^26^ Achilles tendon CSA was measured in transverse at the rupture site if the rupture was distal to the soleus MTJ. The distance from the rupture to the OTJ on the calcaneal bone was measured using skin marks and a tape measure. CSA was measured at the uninjured side on the same distance from the OTJ on the calcaneal bone.^1^ If the site on the uninjured tendon was proximal to the soleus MTJ, CSA was measured right below the soleus MTJ. A measurement tool of the US machine was used to measure CSA and TL. The mean value of 3 calculations was used.^1^

Difference in CSA (measured in cm^2^) was calculated as a ratio (%) of the injured side in relation to the uninjured side (*injured side CSA*/*uninjured side CSA*× 100%). Achilles TE (measured in cm) was calculated as the difference in Achilles TL between the injured and uninjured sides. US screening for deep venous thrombosis (DVT) was conducted as previously described^2^ at 2 and 6 weeks postoperatively.

### Patient-Reported Outcomes

Patient-reported outcome was registered at 12 months by using the validated Achilles tendon Rupture Score (ATRS).^2,18^ ATRS is a self-administered questionnaire consisting of 10 questions, where the maximum score of 100 indicates a complete subjective recovery.^18^

Functional outcomes were assessed at 12 months postoperatively by the heel-rise endurance test (HRET), supervised by 1 experienced physical therapist (S.A.).^2,3,25^ During the HRET, the patient performed heel raises to as high as possible with a straight knee standing on 1 leg on a 10° incline wearing standardized footwear attached to a linear encoder (MuscleLab; Ergotest Technology) after a 5-minute warm-up on a stationary bike and 10 repetitions of heel raises on both legs.^2,25^ The uninjured side was tested first. A standardized tempo of 30 heel raises per minute was used, and the test was terminated when the patient could not maintain the tempo or stopped performing the heel raises.^3^ Total concentric work (TCW; in joules) was obtained from the measurement tool output (TCW = *body weight* [kg] ×*total vertical displacement* [m] × 9.807 [a constant to convert kilopond meters to joules]).^4^ Maximum heel-rise height (HRH_max_) was registered. The limb symmetry index (LSI; ratio [%] of functional performance on the injured relative to the uninjured limb: *injured/uninjured*× 100%) was calculated for TCW and HRH_max_.

### Statistical Analysis

SPSS (Version 27; IBM) was used for all analyses. Standard descriptive statistics were conducted, and all variables were checked for skewness.

Associations between tendon morphology at 6 weeks, 6 months and 12 months and patient outcomes at 12 months were investigated using multiple linear regression. Forward linear regression was used to evaluate if sex, body mass index, time from injury to surgery, duration of surgery, or DVT would increase the model precision in addition to patient age. Final regression analyses were adjusted only for patient age.

The area under the receiver operating characteristic curve (AUC) was calculated for prognostic capacity assessment. For the purposes of AUC analyses, patient outcomes were dichotomized as ATRS >80 vs ≤80, LSI HRH_max_ >80% vs ≤80%, and LSI TCW >80% vs ≤80%. Patients were analyzed as a single group despite postoperative treatment group to increase statistical power. CSA, TE, and long-term patient outcomes for each group separately at different time points have been previously reported by Aufwerber et al.^1^ Tendon CSA was not influenced by EFM at any time point.^1^ Early TE and ATRS were increased in EFM, but at 12 months no differences remained.^1^ Level of significance was set to <.0019 after Bonferroni correction.

## Results

### Tendon Morphology at Different Time Points

Descriptive statistics of Achilles tendon CSA, TL, and TE at different time points are presented in Table 2. The CSA of the injured tendon at 6 weeks was 339% of the uninjured tendon. Mean TE ranged from 1.85 cm at 6 weeks to 1.71 at 12 months (Table 2.). Mean ATRS was 79 (n = 80) in patients who participated in US measurements of tendon morphology at ≥1 time point and 83 (n = 44) in patients who did not participate (*P* = .196).

**Table 2:** Achilles Tendon Morphology of Patients (N = 86) at 6 Weeks, 6 Months, and 12 Months After Surgery^*a*^

Measurement	Time Point		
	6 Weeks	6 Months	12 Months
CSA, cm^2^			
Injured side	1.91 (1.76-2.06)	2.77 (2.60-2.94)	2.10 (2.00-2.21)
Uninjured side	0.58 (0.55-0.61)	0.58 (0.54-0.62)	0.56 (0.53-0.59)
CSA ratio, %^*b*^	339 (311-368)	497 (458-536)	389 (366-413)
	(n = 48)	(n = 60)	(n = 65)
TL, cm			
Injured side	24.2 (23.5-24.8)	24.2 (23.6-24.8)	23.8 (23.3-24.4)
Uninjured side	22.3 (21.7-23.0)	22.4 (21.9-23.0)	22.1 (21.6-22.6)
TE, cm^*c*^	1.85 (1.59-2.10)	1.78 (1.54-2.02)	1.71 (1.49-1.94)
	(n = 57)	(n = 71)	(n = 82)

a Data are presented as mean (95% CI). CSA, cross-sectional area; TE, Achilles tendon elongation; TL, Achilles tendon length.

b Calculated as (*injured side CSA*/*uninjured side CSA*) × 100%.

c Calculated as injured side TL – uninjured side TL.

### Association of Achilles Tendon Morphology With Patient Outcomes

**Figure 3.**
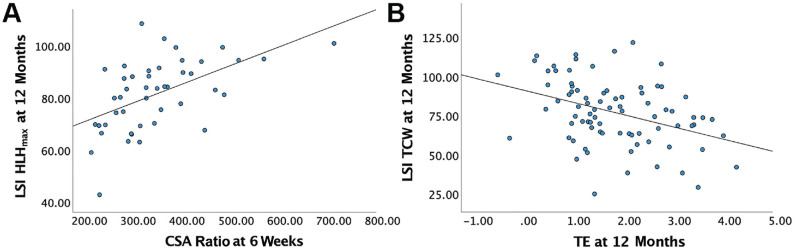
Scatterplots with unadjusted regression lines for (A) CSA ratio at 6 weeks with LSI HRH_max_ and (B) TE at 12 months with LSI TCW. CSA, cross-sectional area; HRH_max_, maximum heel-rise height; LSI, limb symmetry index; TCW, total concentric work; TE, tendon elongation.

Only a larger CSA ratio at 6 weeks was significantly associated with a higher LSI HRH_max_ at 12 months (*R*^2^, 0.35; β, 0.07; *P* < .001) (Table 3, Figure 3). There was no significant association with CSA ratio at 6 months and 12 months with LSI HRH_max_. Only a larger TE at 12 months was significantly associated with lower LSI TCW (*R*^2^, 0.21; β, –7.3; *P* = .001) (Table 3, Figure 3). There was no significant association with TE at 6 weeks and 6 months with LSI TCW.

**Table 3:** Association of Achilles Tendon Morphology at Different Time Points With Patient Outcomes at 12 Months After Surgery^*a*^

Time Point	ATRS		LSI HRH_max_		LSI TCW	
	Crude	Adj	Crude	Adj	Crude	Adj
CSA ratio^*b*^						
6 wk						—
No.	42	—	45	45	45	—
*R*^2^	0.01	—	0.29	0.35	0.05	—
β	−0.02	—	0.07	0.07	−0.04	—
*P*	.462	—	**<.001**	**<.001**	.145	—
6 mo						
No.	56	56	58	58	58	—
*R*^2^	0.09	0.10	0.07	0.17	0.01	—
β	−0.03	−0.04	0.02	0.02	0.01	—
*P*	.021	.021	.039	.110	.567	—
12 mo						
No.	62	—	65	65	65	—
*R*^2^	0.04	—	0.06	0.14	0.03	—
β	−0.03	—	0.03	0.02	0.03	—
*P*	.128	—	.043	.138	.186	—
TE						
6 wk						
No.	51	—	53	—	53	—
*R*^2^	0.004	—	0.01	—	0.006	—
β	−1.1	—	−1.5	—	−1.4	—
*P*	.641	—	.402	—	.568	—
6 mo						
No.	66	—	68	68	68	68
*R*^2^	0.01	—	0.07	0.18	0.09	0.17
β	−1.8	—	−3.2	−2.5	−5.5	−4.6
*P*	.363	—	.033	.800	.016	.038
12 mo						
No.	79	—	82	82	82	82
*R*^2^	0.02	—	0.10	0.17	0.15	0.21
β	−2.1	—	−4.6	−4.1	−7.9	−7.3
*P*	.244	—	.004	.008	**<.001**	**.001**

a Simple linear regression or multiple linear regression adjusted for patient age. Boldface *P* values indicate statistical significance (*P* < .0019 after Bonferroni correction). Dashes indicate areas not applicable. Adj, adjusted; ATRS, Achilles tendon Total Rupture Score; CSA, cross-sectional area; HRH_max_, maximum heel-rise height; LSI, limb symmetry index; TCW, total concentric work; TE, tendon elongation.

b Calculated as (*injured side CSA*/*uninjured side CSA*) × 100%.

A larger tendon CSA ratio at 6 months was associated with higher ATRS (*R*^2^, 0.10; β, –0.04; *P* =.021) (Table 3, Figure 3), a larger TE at 6 months was associated with lower LSI TCW (*R*^2^, 0.17; β, –4.6; *P* = .038) (Table 3, Figure 3), and a larger TE at 12 months was associated with lower LSI HRH_max_ (*R*^2^, 0.17; β, –4.1; *P* = .008) (Table 3, Figure 3) but were not significant after Bonferroni correction. More information is available in Supplemental Figure S1, available online.

### Predictive Capacity

**Figure 4.**
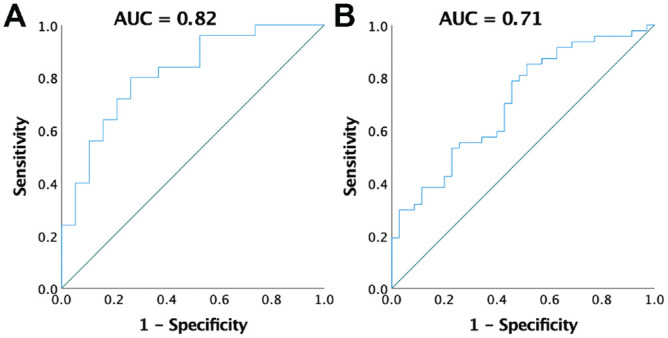
ROC curves with AUC values for (A) CSA ratio at 6 weeks predicting LSI HRH_max_ >80% at 12 months and (B) TE at 12 months predicting LSI TCW ≤80% at 12 months. AUC, area under the ROC curve; CSA, cross-sectional area; HRH_max_, maximum heel-rise height; LSI, limb symmetry index; ROC, receiver operating characteristic; TCW, total concentric work; TE, tendon elongation.

An LSI HRH_max_ >80% was considered an acceptable outcome at 12 months. Larger CSA ratio at 6 weeks had a good clinical prediction of achieving >80% LSI HRH_max_ at 12 months postoperatively (AUC, 0.82; Figure 4A). Greater TE at 12 months had an acceptable diagnostic capacity of achieving ≤80% or less LSI TCW at 12 months postoperatively (AUC, 0.71; Figure 4A). More information is available in Supplemental Figure S2, available online.

## Discussion

The main finding of this study was that a larger Achilles tendon CSA ratio during early healing at 6 weeks after ATR repair exhibited a good clinical prediction of better side symmetry of the HRH_max_ at 12 months postoperatively. More extensive TE at 12 months had acceptable diagnostic capacity of less symmetric TCW performance.

The alterations observed in Achilles tendon CSA (ie, callus size) at different time points in this study are likely to reflect the phases of tendon healing and comparable with values measured by Zellers et al^31^ and Hiramatsu et al.^11^ The observation that Achilles tendon CSA at 6 weeks was significantly associated with a better side symmetry of HRH_max_ at 12 months supports the work of Zellers et al,^31^ which demonstrated that Achilles tendon CSA at 3 months postoperatively was positively associated with functional outcome at 12 months.^31^ In animal models, larger rupture callus and improved tendon stiffness coexist during early healing,^8,14^ implying importance of early proliferation. Based on the current results and earlier studies, it is plausible that a large Achilles tendon CSA during the proliferative phase may reflect an effective healing response with sufficient collagen synthesis capacity. The good predictive capacity^15^ of the Achilles tendon CSA observed at 6 weeks postoperatively supports its usefulness as a biomarker that may have applications in clinical practice and in research to speed up the development of healing-optimizing treatment methods.

Interestingly, at 6 months postoperatively, a larger tendon CSA was associated with poorer patient-reported outcome at 12 months; however, the association was no longer significant after Bonferroni correction. Zellers et al^31^ reported an opposite prediction, which, together with lack of any association with functional outcomes in our data, makes this observation controversial. However, at 6 months, the healing Achilles tendon is in the remodeling phase characterized by increasing type I collagen content, structural modifications to increase the mechanical properties,^29^ and a modest decrease in tendon CSA. An interesting hypothesis to consider is that a larger CSA at this stage could indicate a delay or disturbance in the remodeling phase, leading to inferior tendon properties, but this requires more research. This hypothesis is supported by results from Eliasson et al^7^ that high metabolic activity at 6 months after ATR may be related to poor healing outcome. In a mouse model of Achilles tendinopathy, loading simultaneously decreased CSA and increased material properties,^22^ which is a phenomenon hypothetically also related to postrupture conditions during late remodeling.

The observation at 12 months that the healing Achilles tendon still has a >3 times larger CSA suggests that the remodeling phase is far from over at this stage or that CSA enlargement is permanent, which is corroborated by earlier research suggesting that the tendon never fully recovers.^9,17^ Our findings that the tendon CSA at 12 months was not associated with patient outcomes further strengthens the observation that optimal early healing response may be an essential determinator of good long-term patient outcomes.

The observation that more TE at 12 months was associated with less side symmetry of the TCW at 12 months postoperatively is reasonable regarding tendon biomechanics and is supported by the results of an earlier study.^27^ TE also exhibited acceptable diagnostic capacity for current poor side symmetry of the TCW. However, whether TE exhibits a predictive capacity remains unclear from the present study.

The finding that more TE at 6 months was associated with less side symmetry of the HRH_max_ at 12 months was no longer significant after Bonferroni correction. Olsson et al^20^ reported that heel-rise height, which correlates with TL,^27^ at 6 months predicts patient-reported outcome at 12 months.^20^ Zellers et al^31^ reported that >3 cm TE at 6 months predicted inferior performance in jumping tests at 12 months, but not in the HRET.^31^ Therefore, tendon TE at 6 months may have predictive capacity for patient outcomes at 12 months even though it could not be verified in the current study.

The observation at 6 weeks postoperatively that TE was not associated with long-term patient outcomes complies with earlier evidence that TE increases up to 6 months after which there is only modest shortening or no dynamics.^1,6,12,30,31^ Based on our observations and earlier evidence, we therefore conclude that 6 weeks postoperatively is too early a time point to provide fully useful prognostic information of TE as a biomarker, even though Achilles tendon resting angle is commonly included in the clinical status when lower-leg immobilization is terminated.

### Limitations

The main study limitations were different postoperative regimes, nonstandardized rehabilitation after the initial 6 weeks, only 3 time points studied, and that not all patients were studied at all time points. However, the different postoperative regimes used did not affect either the pattern of tendon CSA^1^ or the patient outcomes at 12 months according to Aufwerber at al.^3^ Aufwerber et al,^3^ however, reported more early TE associated with EFM, but at 12 months TE was similar in the EFM and standard treatment groups.^1^ Nonetheless, since we were interested in evaluating whether tendon morphology predicted patient outcome, a possible mediator effect of different postoperative regimes was deemed acceptable. Tightness of surgical adaptation possibly affecting TE, CSA, and patient outcome was not measured, and it is therefore unclear if surgical technique rather than healing response determines tendon morphology. However, predefined standardized surgical techniques were used. Only surgically treated patients were included, and the generalizability to nonsurgically treated patients is yet to be established. The main benefits of this study are a relatively large sample size, use of US to reliably assess tendon morphology,^28^ and use of patient-reported as well as functional patient outcome measures. Studies on factors modifying tendon CSA and TE are warranted to direct clinical recommendations toward healing-optimizing treatments.

## Conclusion

Greater Achilles tendon CSA seen on US during proliferative healing stage 6 weeks after surgical repair had good clinical prediction for long-term functional patient outcome. TE during the regenerative healing phase 12 months after surgery was predictive of inferior functional outcomes, but assessment of TE at 6 weeks postoperatively was too early to provide useful prognostic information about long-term patient outcomes.

## Supplemental Material

sj-pdf-1-ojs-10.1177_23259671231205326 – Supplemental material for Early Tendon Morphology as a Biomarker of Long-term Patient Outcomes After Surgical Repair of Achilles Tendon Rupture: A Prospective Cohort Study Click here for additional data file. Supplemental material, sj-pdf-1-ojs-10.1177_23259671231205326 for Early Tendon Morphology as a Biomarker of Long-term Patient Outcomes After Surgical Repair of Achilles Tendon Rupture: A Prospective Cohort Study by Annukka Saarensilta, Susanna Aufwerber, Karin Grävare Silbernagel and Paul Ackermann in Orthopaedic Journal of Sports Medicine
