# Supplementary material for: Early Tendon Morphology as a Biomarker of Long-term Patient Outcomes After Surgical Repair of Achilles Tendon Rupture: A Prospective Cohort Study
Source: Orthop J Sports Med. 2023 Nov 6;11(11):23259671231205326. doi: 10.1177/23259671231205326 (PMC10629330; doi:10.1177/23259671231205326)
Supplement: sj-pdf-1-ojs-10.1177_23259671231205326 – Supplemental material for Early Tendon Morphology as a Biomarker of Long-term Patient Outcomes After Surgical Repair of Achilles Tendon Rupture: A Prospective Cohort Study [file sj-pdf-1-ojs-10.1177_23259671231205326.pdf]

Supplemental Material

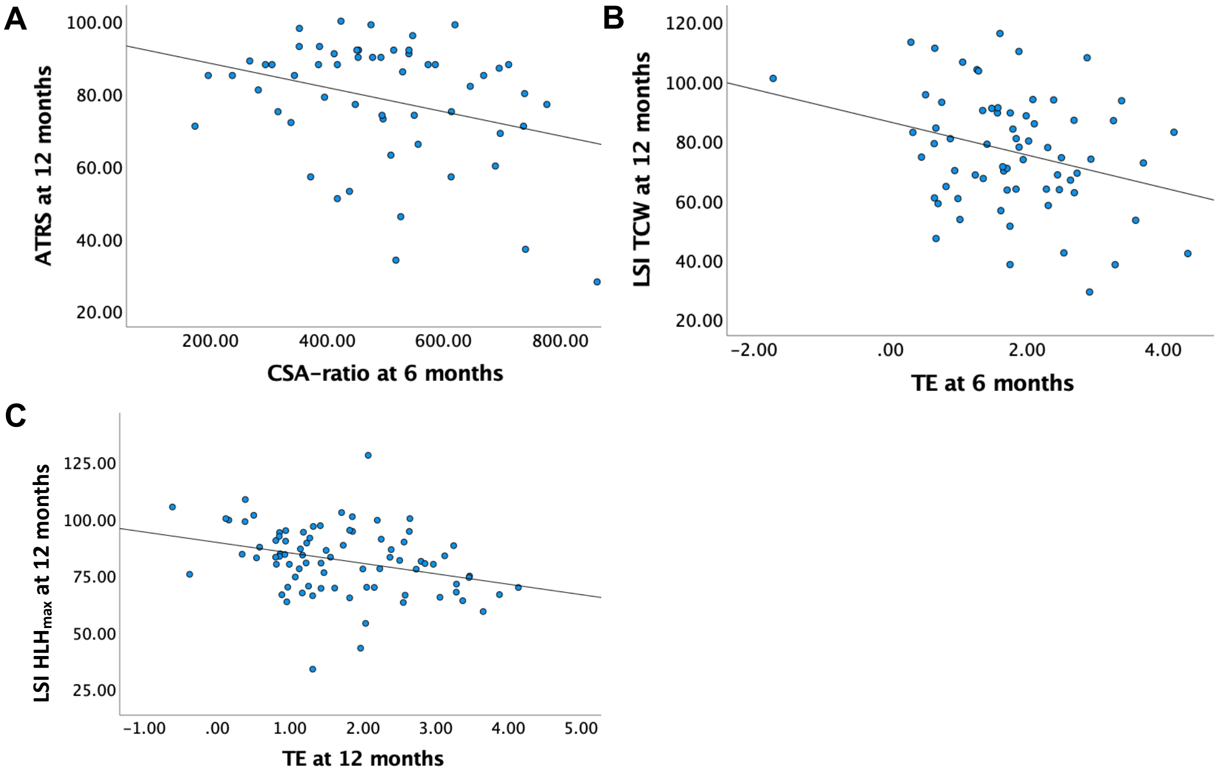

Supplemental Figure S1. Scatter plots with unadjusted regression lines for (A) CSA ratio at 6 months with ATRS, (B) TE at 6 months with LSI TCW, and (C) TE at 12 months with LSI HRH_{max}. ATRS = Achilles tendon Total Rupture Score, TCW = total concentric work, TE = tendon elongation; HLH_{max} = maximum heel-rise height

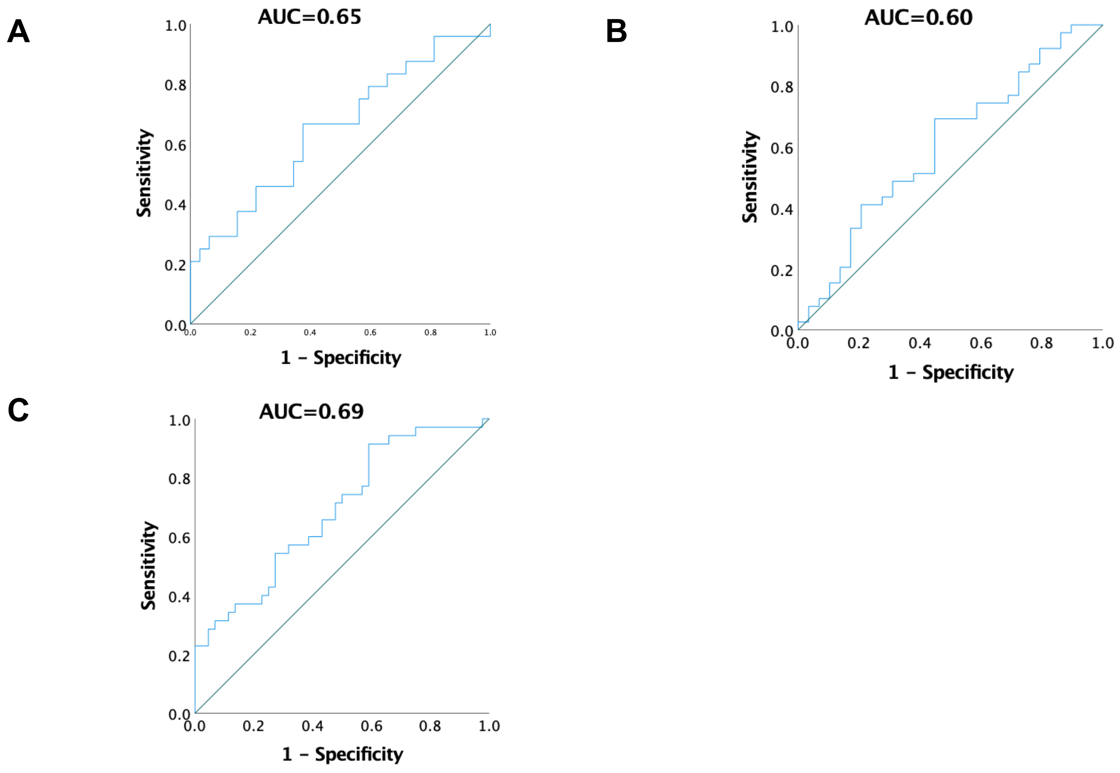

Supplemental Figure S2. ROC curves with AUC values: (A) CSA ratio at 6 months predicting ATRS of ≤80. (B) TE at 6 months predicting LSI TCW of ≤80%. (C) TE at 12 months predicting LSI HLH_{max} of ≤80%. AUC = area under the receiver operating characteristic curve, ATRS=Achilles tendon Total Rupture Score, CSA= cross-sectional area, ROC = receiver operating characteristic, TCW = total concentric work, TE = tendon elongation, LSI = limb symmetry index, HLH_{max} = maximum heel-rise height.
